# Structural Analysis of The OXA-48 Carbapenemase Bound to A “Poor” Carbapenem Substrate, Doripenem

**DOI:** 10.3390/antibiotics8030145

**Published:** 2019-09-11

**Authors:** Krisztina M. Papp-Wallace, Vijay Kumar, Elise T. Zeiser, Scott A. Becka, Focco van den Akker

**Affiliations:** 1 Department of Biochemistry, Case Western Reserve University, Cleveland, OH 44106, USA; vxk107@case.edu; 2 Department of Medicine, Case Western Reserve University, Cleveland, OH 44106, USA; 3 Research Service, Louis Stokes Cleveland VAMC, 10701 East Blvd 151W, Cleveland, OH 44106, USA; elise.zeiser@va.gov (E.T.Z.); scott.becka@va.gov (S.A.B.)

**Keywords:** carbapenemase, structural biology, OXA-48, carbapenem-resistant Enterobacteriaceae

## Abstract

Carbapenem-resistant Enterobacteriaceae are a significant threat to public health, and a major resistance determinant that promotes this phenotype is the production of the OXA-48 carbapenemase. The activity of OXA-48 towards carbapenems is a puzzling phenotype as its hydrolytic activity against doripenem is non-detectable. To probe the mechanistic basis for this observation, we determined the 1.5 Å resolution crystal structure of the deacylation deficient K73A variant of OXA-48 in complex with doripenem. Doripenem is observed in the Δ^1^R and Δ^1^S tautomeric states covalently attached to the catalytic S70 residue. Likely due to positioning of residue Y211, the carboxylate moiety of doripenem is making fewer hydrogen bonding/salt-bridge interactions with R250 compared to previously determined carbapenem OXA structures. Moreover, the hydroxyethyl side chain of doripenem is making van der Waals interactions with a key V120 residue, which likely affects the deacylation rate of doripenem. We hypothesize that positions V120 and Y211 play important roles in the carbapenemase profile of OXA-48. Herein, we provide insights for the further development of the carbapenem class of antibiotics that could render them less effective to hydrolysis by or even inhibit OXA carbapenemases.

## 1. Introduction

**Figure 1:**
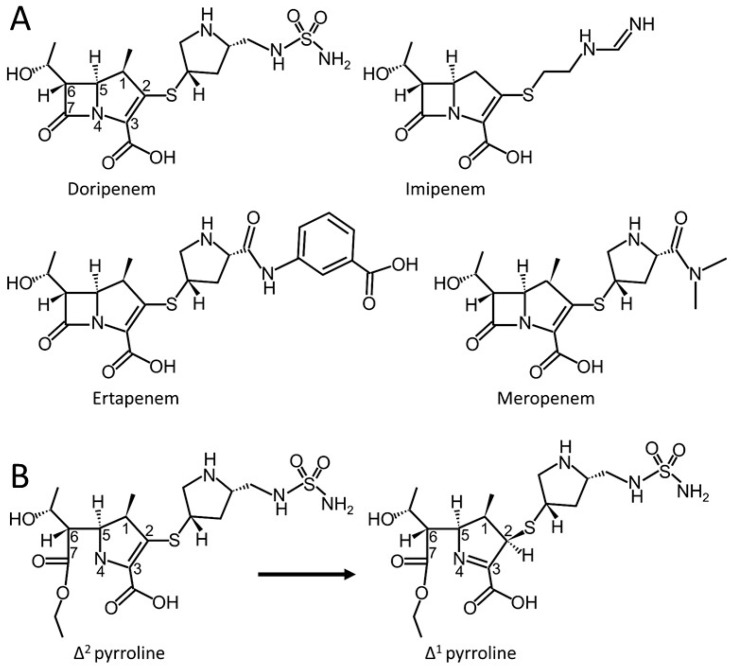
Structures of doripenem, imipenem, ertapenem, and meropenem (**A**) Chemical structures of doripenem, imipenem, ertapenem, and meropenem; the left side of each carbapenem contains the R1 hydroxyethyl side chain that is present in all carbapenems, while the right side of the molecules is denoted as the R2 side chain, which varies in chemistry in different carbapenems. (**B**) Tautomerization states of the acyl intermediate of doripenem in Δ^2^ and Δ^1^R forms.

Carbapenems are a part of a broader class of antibiotics known as β-lactams and they are considered “last resort” antibiotics. They work by inhibiting the penicillin-binding proteins (PBPs), which are the major enzymes involved in bacterial cell wall synthesis. Distinctive to this class of β-lactams is their ability to not only inhibit PBPs, but also serve as inhibitors (i.e., “slow substrates”) for most serine β-lactamases, which are enzymes that confer resistance to β-lactams by hydrolyzing the amide bond of the β-lactam preventing the drug from reaching the PBP [1,2,3,4,5]. Unique characteristics of carbapenems that allow for the inhibition of serine β-lactamases include a R1 hydroxyethyl side chain and the isomerization potential of the pyrroline ring (Figure 1). In many serine β-lactamases, steric hindrance by the hydroxyethyl side chain prevents the approach of the deacylating water molecule as well as interactions with the hydroxyethyl may lower the nucleophilicity of active site residues [6], while tautomerization from the Δ^2^ to Δ^1^ results in a potentially more stable β-lactamase-carbapenem complex [3,7,8]. Additionally, the Δ^1^ tautomer exists in two conformations the Δ^1^S and Δ^1^R (Figure 1), with Δ^1^R possibly being less susceptible to hydrolysis [9]; although a later study indicated that differences in tautomeric states are likely reflective of the active site of the β-lactamase [10]. 

With the use and misuse of carbapenems, resistance to these “last resort” agents has become a global health threat. Carbapenem-resistant gram-negative pathogens are considered priority one critical pathogens by the World Health Organization for the development of novel antibiotics [11]. Resistance to carbapenems is predominantly the result of the expression of carbapenemases, β-lactamases that have acquired the ability to hydrolyze carbapenems [12]. Carbapenemases are either metallo- or serine-based β-lactamase enzymes. Of the serine carbapenemases, the OXA family of carbapenem-hydrolyzing class D β-lactamases (CHDLs) represent a formidable treatment challenge [13]. These CHDLs include OXA-48, which is mostly produced by Enterobacteriaceae, as well as OXA-23, -24/40, -51, and -58 that are primarily expressed by *Acinetobacter* species [14]. 

The mechanisms involved in the turnover of carbapenems have been studied in select CHDLs and structurally compared to their non-CHDL oxacillinase counterparts [14,15,16,17]. Important structural features in CHDLs exist that allow for the conversion of a carbapenem from an inhibitor of serine β-lactamases to a substrate for them. OXA-48 is referred to as the “phantom menace” or “hidden threat” as clinical isolates producing OXA-48 are often difficult to detect, missed by routine diagnostics, and the *bla*_OXA-48_ gene is localized to a plasmid that disseminates rapidly [18,19,20]. Alone, OXA-48 is not a particularly efficient carbapenemase, however, when found in tandem with other resistance determinants such as defective porins and CTX-M-15, an extended-spectrum β-lactamase, carbapenem resistance is more pronounced [21]. Alarmingly, despite imipenem minimum inhibitory concentrations (MIC) that are considered susceptible (0.5 mg/L) by the Clinical Laboratory Standards Institute, imipenem is unable to promote the survival of mice infected with *Klebsiella pneumoniae* producing OXA-48 [22]. The resulting mortality rate in patients with infections caused by OXA-48-producing *Enterobacteriaceae* is 50%; thus, the discovery of novel agents to target these resistant pathogens is critical [23]. 

When comparing different carbapenems, OXA-48 most efficiently hydrolyzes imipenem with a *k*_cat_/*K*_m_ value of 370 mM^−1^s^−1^, whereas hydrolysis of meropenem and ertapenem are appreciably less at 6 mM^−1^s^−1^ and 1 mM^−1^s^−1^, respectively and doripenem turnover is not detectable [17,20,21,24]. Interestingly, the three latter carbapenems all have a methyl at the C1 position in the pyrroline ring whereas the more readily hydrolyzed imipenem lacks such a methyl group. Here, we sought to understand the properties of doripenem that prevent this carbapenem from being hydrolyzed by OXA-48. In addition to differences at the pyrroline C1 position, these carbapenems possess different R2 side chains (Figure 1). The major active site residues in OXA-48 that contribute to β-lactam hydrolysis are the catalytic serine residue, S70 that forms a covalent/acyl bond with the substrates, and K73, which needs to be carbamylated in OXA-β-lactamases to aid in the deacylation reaction involving a water molecule [14,17]. In addition, the β5-β6 loop (residues following Y211) appears to directly contribute to carbapenem hydrolysis, as OXA-48 variants with deletions (e.g., OXA-163) or amino acid substitutions in this region are unable to turnover carbapenems [20,25,26,27]. One exception is the OXA-48 variant OXA-162, which harbors the T213A substitution, which yields increased hydrolysis of doripenem [24].

To probe the molecular basis for carbapenem selectivity, we determined the crystal structure of the K73A deacylation-deficient mutant of OXA-48 in complex with doripenem at 1.5 Å resolution. Additionally, we carried out detailed structural comparisons with previously determined CHDL and non-CHDL OXA structures bound to doripenem including a very recently published structural investigation of multiple carbapenems binding to wild-type OXA-48 obtained at slightly lower resolutions [28]. Identifying the factor(s) that hinders doripenem catalysis by OXA-48 will provide medicinal chemists with pivotal insights needed to design novel therapeutics.

## 2. Materials and Methods

### 2.1. Cloning of bla_OXA-48 K73A_

The gene encoding the OXA-48 K73A variant was cloned into the pET-24a(+) vector using the following methods. The *bla*_OXA-48 K73A_ gene was synthesized and cloned into pBC SK(−) by Celtek Bioscience. Subsequently, *bla*_OXA-48 K73A_ without the codons coding for the signal peptide was amplified via PCR from pBC SK(−) *bla*_OXA-48 K73A_ and subcloned into the pCR 2.1-TOPO vector. The resulting pCR 2.1-TOPO *bla*_OXA-48 K73A_ plasmid and the pET-24a(+) vector were digested with NdeI and HindIII restriction enzymes and ligated with the T4 DNA ligase. The pET-24a(+) *bla*_OXA-48 K73A_ was sequence verified (Molecular Cloning Laboratories) and transformed into *Escherichia coli* BL21 DE3 cells for protein purification.

### 2.2. Expression and Purification of the OXA-48 K73A Variant

*E*. *coli* BL21 DE3 cells carrying the pET-24a(+) *bla*_OXA-48 K73A_ were grown in superoptimal broth to log phase and induced with 500 µM isopropyl β-D-1-thiogalactopyranoside for three hours before harvesting. Pellets were resuspended in 50 mM Tris-chloride (Cl) at pH 7.4 with 1 mM magnesium sulfate. Pellets were lysed with 40 mg/L lysozyme and 1.0 U/mL benzonase nuclease (Novagen) was added to digest the nucleic acids for 45 min. 2.0 mM ethylenediaminetetraacetic acid (EDTA) was added to complete the lysis. The lysed cells were centrifuged at 12,000 rpm for 10 min to remove the cellular debris. The supernatant was further enriched for β-lactamase using preparative isoelectric focusing (pIEF). A Sephadex G100 gel matrix and commercially prepared ampholines (pH 3.5–10.0) were used in the pIEF gel. The pIEF gel was run overnight at 4 °C at a constant power of 8 W on a Multiphor II isoelectric focusing apparatus (Amersham Pharmacia Biotech). After the pIEF gel was run overnight, areas of the gel demonstrating β-lactamase activity by nitrocefin overlay were cut from the gel and eluted with cold 50 mM sodium phosphate buffer pH 7.2. The fractions were run on using sodium-dodecyl sulfate polyacrylamide gel electrophoresis (SDS-PAGE) and stained with Coomassie Brilliant Blue R250. The fractions with the highest protein yield were pooled together and dialyzed overnight in 50 mM sodium phosphate buffer pH 7.2 at 4 °C. The dialyzed sample was collected and a Sephadex 16/60 gel filtration chromatography column on an ÄKTA purifier 10 (GE Life Sciences) was used for polishing steps. SDS-PAGE was used to assess the purity of the fractions. Furthermore, the molecular weight and purity of the protein was evaluated using electrospray ionization mass spectrometry, as previously described [29]. The final protein concentration was determined by measuring the absorbance at 280 nm.

### 2.3. Crystallography

Prior to crystallization, the OXA-48 K73A variant was treated with doripenem (1:20 molar ratio) in 50 mM phosphate buffer pH 7.2 at 4 °C for two hours; the reacted protein was subsequently buffer exchanged on a PD-10 column with 50 mM Tris pH 7.6. Crystals were grown using the sitting drop method with the reservoir containing 0.1 M HEPES pH 7.5–8.5, 4–10% PEG4000, and 8% 1-Butanol. The crystal for data collection was cryo-protected with mother liquor and 12% ethylene glycol before freezing in liquid nitrogen. Diffraction data were collected at the SSRL beamline 9-2 and processed using XDS/autoxds [30]. The diffraction resolution of the data set was 1.5 Å (see Table 1 for additional data collection statistics). The structure was solved using molecular replacement utilizing the apo OXA-48 structure (PDBid: 4S2P) as a search model with the program MOLREP [31]. Refinement was carried out using REFMAC 5 and model building was done with the program COOT [32]. There were two OXA-48 molecules (molecule A and molecule B) in the asymmetric unit with each of them having a covalently bound doripenem molecule attached to the catalytic S70. Two chloride ions were located and refined as well with one of them located in the active site (in molecule B). In addition, a HEPES buffer molecule was observed and refined in close proximity to one of the active sites. The final R/R_free_ was 15.12/17.15% (see Table 1 for additional refinement statistics). The coordinates and structure factors have been deposited with the Protein Data Bank (PDBid 6PXX). The figures were generated using Pymol (www.pymol.org).

## 3. Results and Discussion

### 3.1. Summary of the OXA-48 K73A-Doripenem Crystal Structure

**Figure 2:**
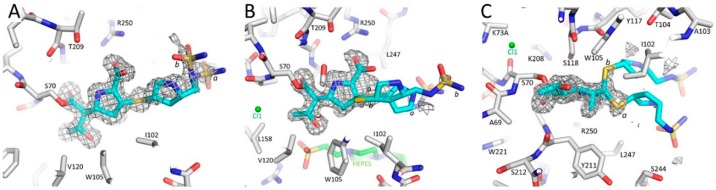
Electron density of doripenem bound in the active site of OXA-48 K73A. (**A**) Unbiased |Fo|-|Fc| electron density difference map of the active site of molecule A of OXA-48 K73 revealing the presence of a covalently bound doripenem (with cyan carbon atoms) attached to S70 (density contoured at 3 σ level). The sulfonamide moiety was observed in two conformations (labeled *a* and *b*). (**B**) Same as in (**A**) but for molecule B of OXA-48 K73A. The tail of doripenem attached to the 2 position of the pyrrolidine ring is observed in two conformations labeled *a* and *b* with occupancies of 0.7 and 0.3, respectively (difference density contoured at 2.75 σ level). (**C**) Same as in (**B**) but rotated ~90° to show the two conformations more clearly.

**Figure 3:**
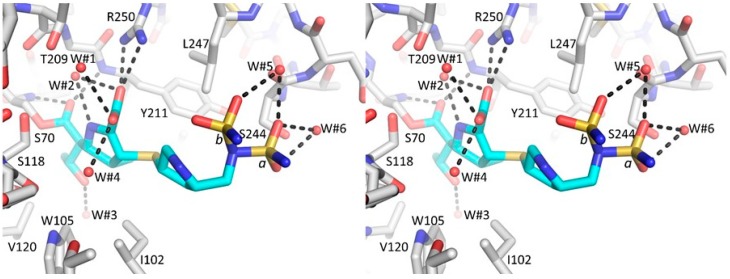
Stereo diagram of doripenem interactions in the active site of OXA-48 K73A molecule A. Hydrogen bonds are depicted as dashed black lines. Interacting water molecules are shown as red spheres.

**Figure 4:**
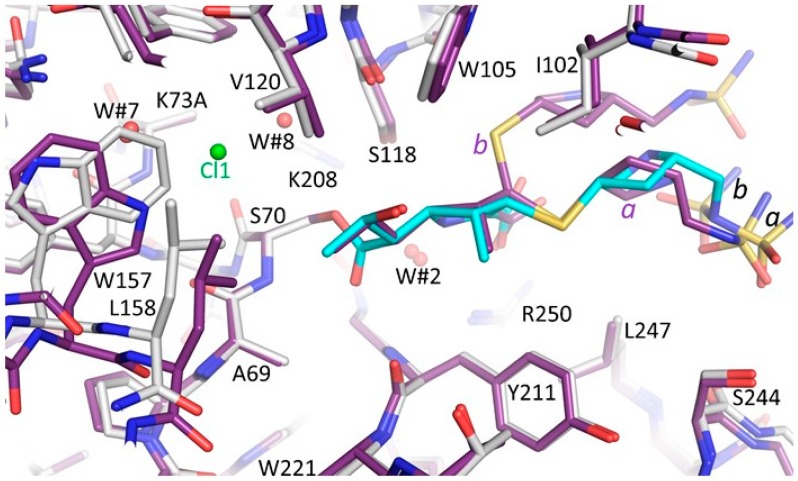
Superposition of doripenem-bound molecules A and B of OXA-48 K73A. Molecules A and B are colored with white and magenta, respectively, carbon atoms; doripenem bound to molecule A is colored cyan. The active site of molecule B has a chloride ion bound (colored green, labeled ‘Cl1’).

We obtained a 1.5 Å resolution data set of the OXA-48 K73A variant co-crystallized with doripenem with two OXA-48 molecules (molecule A and B) in the asymmetric unit. After initial refinement with just the protein coordinates, strong difference density was apparent for the doripenem in both OXA-48 molecules (Figure 2). Doripenem in molecule A was more ordered compared to that in molecule B. In molecule A, the sulfonamide moiety at the end of doripenem’s R2 side chain had weaker density as it was observed in two conformations (*a* and *b*) (Figure 2A and Figure 3); these conformations were refined at 0.5 occupancy each. In molecule A, doripenem’s sulfur atom adjacent to the R2 pyrrolidine ring is in the R-configuration (Δ^1^R). In molecule B, the entire tail attached to the C2 position of the core pyrrolidine of doripenem was less ordered and is in two conformations (*a* and *b*); these tails were refined with occupancies of 0.7 and 0.3, respectively (Figure 2B,C). There was density for the sulfur atom of the R2 side chain in both the R- and S- configurations (*a* (Δ^1^R) and *b* (Δ^1^S)) (Figure 2C). The K73A amino acid substitution was clearly observed in the electron density. In molecule B, the cavity resulting from this mutation was occupied, in part, by a full occupied chloride ion (Figure 2B,C, and Figure 3); in molecule A, the W157 adopts a different conformation that is made possible by the K73A cavity-introducing mutation (Figure 4). Overall, the protein structures of molecule A and B are very similar with a root-mean-square-deviation (r.m.s.d.) of 0.81 Å for 242 Cα atoms. The most significant differences between molecules A and B are observed in the small helix containing W157 and L158 (Figure 4).

### 3.2. Major Interactions Observed Between Doripenem and Active Site Residues

For the most part, the interactions between doripenem and the active site residues of molecule A and B are very similar. There is a covalent (acyl) bond between doripenem and S70 (Figure 3 and Figure 4). The β-lactam carbonyl oxygen occupies the nearby oxyanion hole comprised of backbone nitrogen atoms of S70 and Y211 (Figure 3 and Figure 4). One of the oxygens of the carboxylate moiety of doripenem is interacting with the side chain of R250 thus forming a salt bridge (Figure 3). There is also a nearby water molecule (W#2) making interactions with this carboxylate group and the amine moiety of the core pyrroline ring (Figure 3; W#2); this water also forms hydrogen bonds with R250 and T209. Moreover, doripenem makes hydrogen bonds with a few additional nearby water molecules (Figure 3). The methyl moiety attached to the C1 position of the core pyrroline ring is making van der Waals interactions with the CD2 atoms of Y211 (3.8 Å distance for both doripenem molecules) (Figure 3 and Figure 4). Additional van der Waals interactions of the hydrophobic part of the pyrroline ring involve residue I102 of the 100-109 loop (Figure 3 and Figure 4). The hydroxyethyl moiety is in the same orientation in both doripenem molecules (Figure 4); this moiety is not making direct hydrogen bonds with the protein but is making van der Waals interactions with residues Y105, V120, and L158. In molecule B, the hydroxyethyl moiety is making a hydrogen bond with an adjacent HEPES buffer molecule (Figure 2B). 

### 3.3. Comparison of the OXA-48 K73A-Doripenem Structure to the Wild-Type OXA-48-Doripenem and Wild-Type OXA-48-Imipenem Structures

The structures of OXA-48 complexed to the carbapenems, imipenem, and doripenem, were recently determined using crystal soaking at slightly lower resolutions of 1.95 and 1.9 Å, respectively (PDBid codes: 5QB4 and 6P9C) [28,33]. These OXA-48 crystallographic studies were carried out without a deacylation-deficient substitution that would slow down the deacylation rate. A benefit of not using a deacylation-deficient variant for such structural efforts is that the resulting complex might yield a more natural snapshot of events in the active site as the enzyme is in its native form. A disadvantage is that the resulting density could be difficult to interpret as it might not capture a single inhibitory species since the enzyme is active with rounds of non-synchronized turnover events taking place throughout the crystal. We, therefore, inspected both these structures and structure factors and carried out a 10-cycle Refmac refinement of both structures without the carbapenem ligands present to remove potential phase bias prior to calculating the unbiased electron density different maps. For the wild-type OXA-48-doripenem structure, we also calculated a POLDER omit map [34] similar as in [28], yet still with the ligand removed in a 10-cycle Refmac refinement prior to the map calculation. The resulting unbiased electron density maps contoured at 3 σ showed poor carbapenem electron density in both doripenem and imipenem structures in all non-crystallographically related OXA-48 molecules (Supplementary Materials Figure S1). The omit density only suggests that S70 is covalently bonded to the carbapenem carbonyl carbon and that the carbonyl oxygen is in the oxyanion hole. Density for the pyrroline ring is not convincing with a lack of connected density to the carbonyl moiety and towards and including the ring substituents. The only conclusion one can, therefore, draw from these carbapenem wild-type OXA-48 structures is that these ligands are very disordered when bound to S70, either statistically, dynamically, or as different conformers and/or different inhibitory species. This itself is still useful information for understanding the mechanism of OXA-48 towards these carbapenems.

### 3.4. Comparison of the OXA-48 K73A-Doripenem Structure to An OXA-51 CHDL Variant Bound to Doripenem 

**Figure 5:**
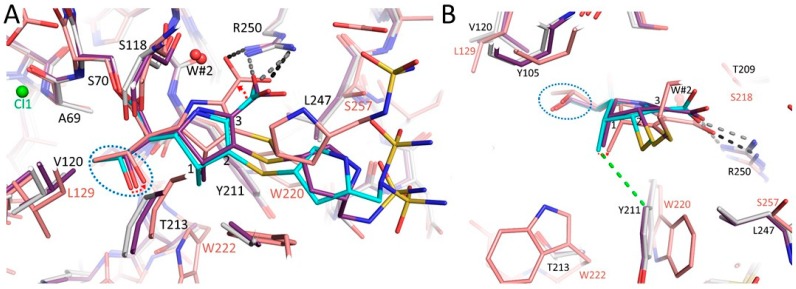
Superposition of the doripenem-bound complexes of OXA-51 I129L/K83D and OXA-48 K73A. (**A**) OXA-51 I129L/K83D, OXA-48 K73A molecules A and B are superpositioned. Carbon atoms for the OXA-51 complex are colored light pink, molecules A and B of OXA-48 K73A are colored as in Figure 3. Hydrogen bonds between the carboxyl moiety and R250 in the OXA-51 I129L/K83D structure are depicted as black dashed lines; hydrogen bonds between doripenem and that same R250 in the OXA-48 K73A molecule A structure are shown as grey dashed lines. The hydroxyethyl moiety of doripenem is highlighted by a blue dashed oval line; positions 1, 2, and 3 on the pyrrolidine ring are numbered. The rotation needed for doripenem’s pyrrolidine ring when bound to OXA-48 to achieve the binding mode as when bound to OXA-51 I129L/K83D is depicted by a small red dashed arrow. (**B**) Rotated such that view is perpendicular to that in (**A**). Van der Waals interaction between the methyl moiety of doripenem and Y211 in the OXA-48 K73A molecule A structure is depicted as a green dashed line.

The OXA-51 I129L/K83D variant bound to doripenem (PDBid: 5L2F) was compared to the OXA-48 K73A-doripenem structure; the K83D amino acid substitution creates a deacylation-deficient mutant and K83 corresponds to residue K73 in OXA-48 (Figure 5). This structure is noteworthy as the I129L substitution (the third residue in the SXV/I conserved motif of OXA-β-lactamases) increases the catalytic efficiency of OXA-51 towards carbapenems; the OXA-51 I129L variant possesses a *k*_cat_/*K*_m_ value of 1400 mM^−1^s^−1^ for doripenem compared to 16 mM^−1^s^−1^ for wild-type OXA-51 (Supplementary Materials Figure S2) [16,35]. The substitution from isoleucine to leucine was found to relieve the steric clash between I129 and the hydroxyethyl of carbapenems, accelerating turnover [35]. Correspondingly, the pyrroline ring of doripenem in the OXA-51 I129L/K83D complex is in the ∆^2^ tautomer state with the β-lactam carbonyl in the oxyanion hole formed by the backbone nitrogen atoms of S80 and W220. In the OXA-48 K73A-doripenem structure, V120 (the analogous residue to I129) adopts a different rotamer conformation and makes van der Waals interactions by pointing one of its CH_3_ groups towards the hydroxyethyl moiety of doripenem. Thus, like I129 in OXA-51, V120 may impede doripenem turnover by OXA-48. Further comparing the OXA-51 I129L/K83D variant-doripenem to the OXA-48 K73A variant-doripenem, an additional difference is evident: The pyrroline ring is rotated in the OXA-51 I129L/K83D complex such that the carboxylate moiety of doripenem is interacting with the R260 (equivalent to R250 in OXA-48) guanidinium side chain using both the carboxylate oxygen atoms (Figure 5). A possible reason for why doripenem is not able to make the hydrogen bonds utilizing both its carboxylate oxygens with the guanidinium side chain of R250 in OXA-48 is that the methyl moiety attached to C1 of the pyrroline ring is in a 3.8 Å distance van der Waals interaction with Y211 (Figure 5B). The needed rotation would bring this methyl moiety to 3.4 Å distance with the Y211 CD2 atom which could lead to (minor) steric hindrance. An alternative although similar explanation is that the adjacent sulfur, being in the Δ^1^R tautomer in molecule A, is at 4.1 Å from the Y211 side chain; the needed rotation might bring this sulfur potentially sterically too close to Y211. Note that in molecule B, the doripenem also has a partial Δ^1^S tautomer which would likely not sterically interact with Y211. In concordance with this postulated role of Y211 influencing the binding of doripenem to OXA-48, for a OXA-48 variant, T213A (in OXA-162), yields increased hydrolysis of doripenem [24]. The *k*_cat_ increased from non-detectable for OXA-48 to 1.38 s^−1^ for OXA-162. It is the T213 side chain that wedges against the Y211 side chain (Figure 4 and Figure 5B) and a substitution to Ala will likely allow Y211 to tilt/shift. Such a Y211 movement could permit this variant to form the proper doripenem interactions with R250 to yield the higher activity towards doripenem as a substrate [24]. Note that this T213A mutant increased the *k*_cat_ for carbapenems with a C1 methyl moiety, ertapenem, doripenem, and meropenem, much more significantly than imipenem which does not have this C1 methyl group [24]. This suggests that residues Y211 and T213 have a strong impact in allowing OXA-48 to preferentially hydrolyze carbapenems without the C1 methyl group.

### 3.5. Comparisons of OXA-48 K73A-Doripenem Structure to OXA-23 and OXA-24/40 CHDL Variants Bound to Doripenem

Additional structural comparisons were carried out with other CHDL-doripenem complexes, OXA-239 (a variant of OXA-23) and OXA-24/40 [15,36]. Both these OXAs possess a “hydrophobic bridge” that narrows the active site significantly (comprised of OXA-23 residues: F110 and M221; OXA-24 residues: Y112 and M223); this bridge is not present in OXA-48. Amino acid substitutions in the hydrophobic bridge of OXA-23 were found to negatively impact the affinity for carbapenems, the *k*_cat_, however, was less affected [10]. Doripenem is covalently attached to the catalytic serine in both structures and utilizes both its carboxylated oxygens to interact with arginine (R259 in OXA-239 and R260 in OXA-24/40) corresponding to residue R250 of OXA-48, as was discussed above for OXA-51. 

OXA-239 possesses an extended profile toward late-generation cephalosporins and aztreonam and a K82D substitution was generated to obtain a deacylation deficient variant in complex with doripenem (PDBid: 5WI7). The wild-type OXA-23 CHDL hydrolyzes doripenem well with a *k*_cat_/*K*_m_ value of 1,600 mM^−1^s^−1^; whereas OXA-239’s catalytic efficiency is ~3-fold lower at 480 mM^−1^s^−1^ [37]. Regarding the OXA-239 complex, the tautomeric state of doripenem could not be elucidated as both Δ^1^ and Δ^2^ isomers equally fit the electron density [36]. The same group also determined the structure of OXA-239 complexed with imipenem and observed the Δ^1^ tautomer for this carbapenem [36]. The side chain of V128 in OXA-239 K82D is pointing away from the active site and the hydroxyethyl side chain of doripenem, unlike V120 in the OXA-48 K73A-doripenem structure. 

The OXA-48 K73A-doripenem structure was also compared in detail to OXA-24/40. The wild type OXA-24/40 enzyme robustly hydrolyzes doripenem with a *k*_cat_/*K*_m_ value of 3,100 mM^−1^s^−1^ [37]. To obtain the complex with doripenem, the OXA-24/40 K84D (PDBid: 3PAE) and V130D (PDBid: 3PAG) deacylation deficient variants were constructed; these residues correspond to K73 and V120 of OXA-48, respectively [15]. In both OXA-24/40 variant structures, the doripenem was in the Δ^2^ tautomer with the β-lactam carbonyl located in the oxyanion hole (backbone amides of S81 and W221), thus is primed for deacylation [15]. The Δ^2^ tautomeric state was suggested to be the result of the reduced flexibility of the R2 side chain of doripenem due to the presence of a hydrophobic bridge between Y112 and M223 in OXA-24/40 [15]. The positioning of the hydroxyethyl side chain in the OXA-24/40 V130D structure is away from K83, thus allowing access of the deacylating water molecule with the methyl of the hydroxyethyl stabilized through a van der Waals interaction with L168 [15]. Moreover, V130 is positioned away from the hydroxyethyl in the OXA-24/40 K83D complex and V130 is stabilizing K83, thus allowing for doripenem turnover [15,35]. This observation is in direct contrast to OXA-48 where the V120 forms van der Waals interactions with the hydroxyethyl side chain of doripenem. 

**Figure 6:**
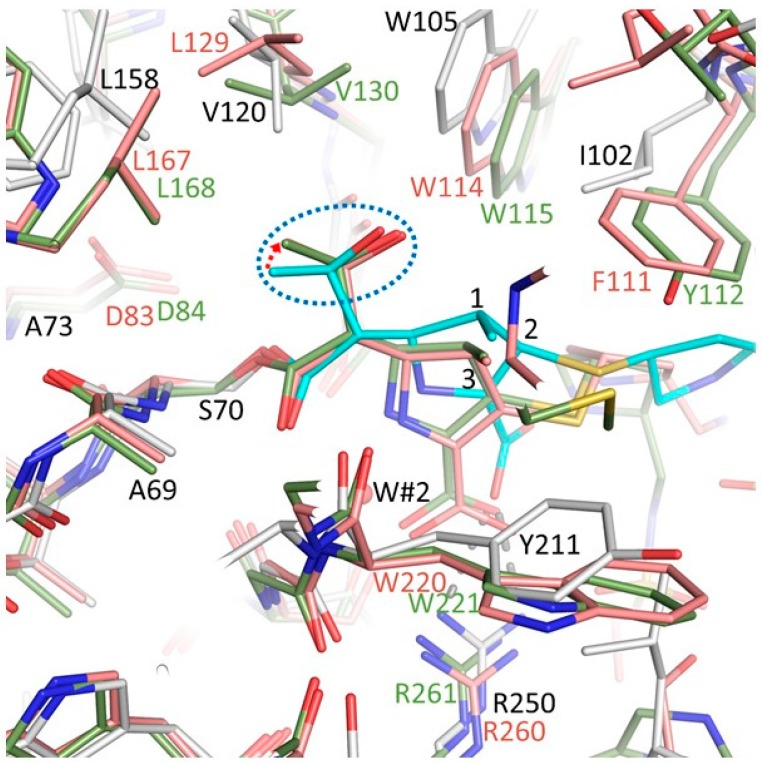
Superposition of the doripenem-bound structures of OXA-24/40 K84D, OXA-51 I129L/K83D, and OXA-48 K73A. Carbon atoms for the OXA-24/40 K84D doripenem complex are colored green; molecule A of OXA-48 K73A and OXA-51 I129L/K83D are colored as in Figure 5. The hydroxyethyl moiety of the doripenem’s pyrrolidine ring atom numbering and rotation are shown as in Figure 5. The superpositioning involved the same set of 25 Cα atoms as in Figure 5 onto their equivalent residues in the OXA-24/40 K84D complex. Except for S70 and A71, all residues of their respective OXAs are labeled and color-coded.

A superposition of complexes of OXA-24/40 K84D, OXA-48 K73A, and OXA-51 I129L/K83D highlights the different pyrroline orientation and different doripenem carboxylate position in OXA-48 compared to the other two OXAs (Figure 6). Because of this different pyrroline orientation, the position of the hydroxyethyl of doripenem in the OXA-48 structure is closer to residue K73A compared to the other two structures. Residue V120 in OXA-48 adopts a different position, compared to the other two OXAs, as mentioned above, having one of its CH_3_ groups point to the hydroxyethyl group of doripenem (Figure 6). These observations could be important as contributing factors to carbapenem turnover by OXA-24/40 including the positioning of the hydroxyethyl side chain away from K83, thus allowing access of the deacylating water molecule with the methyl of the hydroxyethyl stabilized through a van der Waals interaction with L168 [15]. As noted above, the I129L substitution in OXA-51, equivalent to OXA-48 residue V120, leads to a drastic increase in carbapenem hydrolysis [16,35]; the position of L129 is more distant from the hydroxyethyl moiety of doripenem compared to V120 in OXA-48 (Figure 6). Note that the V120 conformation in OXA-48 K73A doripenem is also observed in other OXA-48 structures [17,38] indicating that this conformation is not doripenem-dependent. Therefore, this V120 conformation does not necessarily limit hydrolysis of other β-lactam substrates; however, only the carbapenem class of β-lactams contain a hydroxyethyl R1 side chain. 

An additional observation is that the C1 methyl of doripenem is in 3.2 Å van der Waals interactions with the oxygen of the hydroxyethyl moiety (Figure 6); such distance could impede some rotational freedom for this hydroxyethyl group possibly also contributing, in part, to the overall lower *k_cat_* for carbapenems with a C1 methyl moiety. In addition to these differences, another key difference between OXA-48 and the other two OXAs is that OXA-48 has a Tyr at position 211 whereas the other two have a Trp residue that is orientated away from the pyrroline ring (Figure 6). The presence of this Tyr residue being in close proximity to the C1 methyl could make OXA-48 have decreased activity towards C1 methyl containing carbapenems. The above structural analyses for related class D enzymes point to a key role for OXA-48 residue V120 and the region around it in the deacylation reaction. Furthermore, that the corresponding V130D substitution in OXA-24 was used to obtain a deacylation deficient variant for structural studies, further strengthens the likely role for this residue in the deacylation step as also noted for OXA-51 above. 

### 3.6. Comparisons of OXA-48 K73A-Doripenem Structure to OXA-1, a Non-CHDL Bound to Doripenem

We also analyzed the doripenem OXA-1 complex (PDBid: 3ISG), as OXA-1 does not hydrolyze carbapenems [39]. This OXA possesses many additional active site differences compared to OXA-48 including lack of R250 [39]. Features worth mentioning include that the doripenem molecule is in the Δ^1^R tautomeric state in OXA-1 and the hydroxyethyl side chain of doripenem packs against V117, which is analogous to the interactions between V120 of OXA-48 and the doripenem hydroxyethyl side chain. Amino acid substitutions at V117 were found to alter the spectrum of OXA-1, the substitution of positively charged residues lysine and arginine can maintain significant activity of OXA-1 [40]. The inability of OXA-1 to hydrolyze doripenem was attributed to a lack of an appropriately positioned deacylation water molecule due to hydrogen bonding interactions between the carboxylated K70 of OXA-1 and the hydroxyethyl side chain of doripenem [39]. 

### 3.7. So Why is OXA-48 Unable to Hydrolyze Doripenem? 

Our structural results and comparisons point to a key role for two OXA-48 active site regions that could contribute to its poor activity against doripenem and other carbapenems harboring a C1 methyl at the pyrroline ring. First, residue Y211 is in van der Waals contact with the C1 methyl of doripenem. This van der Waals contact might prevent the carboxylate moiety of doripenem from making stronger interactions with R250; Y211 is not present in the other OXAs discussed above. Second, residue V120 is in a distinct conformation and in close contact with the hydroxyethyl moiety of doripenem, rotation of this doripenem moiety has been postulated to be critical for allowing the deacylation water in the vicinity of K73 to attack the carbonyl carbon atom prior to deacylation [17]. The V120 conformation has been observed in other OXA-48 structures indicating that this conformation itself does not prevent OXA-48 from being an active β-lactamase for other β-lactam substrates. Additionally, previously published carbapenem complexes of doripenem and imipenem with wild-type OXA-48 reveal no well-ordered complexes despite that OXA-48 does not even hydrolyze doripenem; this indicates that multiple conformations/states are occurring for both C1 methyl and non-C1 methyl containing carbapenems. It could well be that the conformation needed for a deacylation water to productively approach the acylated carbapenem is very different from what has previously been observed interacting with R250. It is obvious that the reaction mechanism of OXAs is very complex. For instance, the initial relatively simple steps of a Michaelis-Menten complex formation and acylation are followed by subsequent steps that can take place simultaneously yet likely occur at different rates. These include possible tautomerization from Δ^2^ to Δ^1^R and Δ^1^S, torsional rotation of the hydroxyethyl moiety, deacylation water movement, deacylation, possible de- and carbamylation of K73. These time-dependent events are investigated by different methods and even different crystallographic techniques such as varying soaking times or co-crystallization making it therefore difficult to tease out what really are the rate-limiting steps. Previously, a significant amount of emphasis had been put on tautomerization states and its potential impact on turnover in a variety of different and sometimes distantly related serine β-lactamases but a recent study involving OXA-23 suggested tautomerization to have less of an impact, at least in OXA-23 [10]. Despite all these caveats and uncertainties, our structural results point to an importantrole for OXA-48 residues Y211 and V120 for the poor activity of C1-methyl containing carbapenems and doripenem in particular. 

## 4. Conclusions

The crystal structure of OXA-48 K73A in complex with doripenem was determined to probe the lack of observed catalytic activity of this carbapenemase towards doripenem. There appears to be a delicate balance in the positioning of select active site residues (V120 and Y211) as well as the interactions of these residues with carbapenem moieties that influence whether these drugs are hydrolyzed. Due to the Y211 side chain likely indirectly limiting the carboxylate interactions with the R250 side chain, a novel slightly rotated binding mode with different carboxylate interactions for doripenem was observed in the OXA-48 K73A-doripenem structure. In addition, the rotated doripenem yields different interactions between the hydroxyethyl moiety of doripenem and the active site. Comparisons of this structure to other OXA-doripenem complexes pointed to the V120 residue found in the SXV/I motif of OXA enzymes; the complementing residues to in OXA-1, OXA-23, OXA-24/40, and OXA-51 are V117, V128, V130, and I129, respectively. This residue may play a pivotal role in carbapenem turnover. In enzymes, OXA-1, OXA-48, and OXA-51, where doripenem turnover is not evident, we hypothesize that this residue packs against the hydroxyethyl side chain likely stalling hydrolysis. Conversely, in OXA-239 (OXA-23-family) and OXA-24/40 variants, this residue points away from the active site and in OXA-24/40 variant, V130 also supports the carboxylated K83 residue to promote hydrolysis and the V130D variant is deacylation deficient [15]. Moreover, an OXA-48 variant, OXA-519 was found in a clinical isolate of *Klebsiella pneumoniae*, OXA-519 possesses a V120L amino acid substitution and demonstrates enhanced turnover of meropenem and ertapenem; doripenem was not tested [41]. In the analogous position of OXA-51, the amino acid substitution (I129L) was also originally described in a clinical isolate, further supporting the role of this site in the evolution of enhanced carbapenemase activity [35,42]. These observations could be the structural basis for the limited hydrolytic activity of OXA-48 towards doripenem. The hydroxyethyl side chain is a critical component of carbapenems to prevent their degradation by maintaining their inhibitory activity against β-lactamases. Further development of this side chain by medicinal chemists may lead to the development of novel carbapenems that could be resistant to deacylation by CHDLs.

## Figures and Tables

**Table 1:** Data collection and refinement statistics for the OXA-48 K73A doripenem complex.

Data Collection	OXA-48 K73A Doripenem Complex
Space group	P2_1_2_1_2
Unit cell dimensions (Å, °)	105.77 125.09 45.12 90 90 90
Wavelength (Å)	0.97946
Resolution (Å)	40.38-1.50 (1.53-1.50)
Redundancy	13.2 (12.0)
Unique reflections	96,537 (4,704)
<I>/<σ(I)>	19.6 (5.0)
Mn(I) half-set correlation CC(1/2)	0.99 (0.96)
R_merge_ (%)	7.7 (48.5)
Completeness (%)	99.8 (99.7)
**Refinement**	
Resolution range (Å)	40.42-1.50
R-factor (%)	15.1
R*_free_* (%)	17.2
Estimated coordinate error ESU from R_free_ (Å)	0.06
Number of protein atoms	4030 (2 molecules in the asymmetric unit)
Number of water molecules	603
Ligands (number of atoms)	2 doripenem (28 atoms each), 1 partial HEPES molecule (15 atoms), 2 chloride ions, 5 ethylene glycol molecules (4 atoms each)
Real-space CC of ligands	
Doripenem, mol A; conformation 1, 2	0.96, 0.96
Doripenem mol B; conformation 1, 2	0.91, 0.92
HEPES	0.94
Chloride ions	0.99, 0.99
RMSD deviation from ideality	
Bond length (Å)	0.011
Bond angles (°)	1.74
Ramachandran plot statistics (%)	
Preferred regions	96.4
Allowed regions	3.6
Outliers	0.0

High resolution shell values are in parenthesis.

## Supplementary Materials

The following are available online at https://www.mdpi.com/2079-6382/8/3/145/s1, Figure S1: Electron density maps of the active sites of wild-type OXA-48 with doripenem and imipenem bound. To remove ligand bias prior to the map calculation, a 10-cycle refmac crystallographic refinement was carried out with the ligands removed from the coordinates., Figure S2: Multiple sequence alignment using Clustal Ω using wild-type enzymes of OXA-1, OXA-23, OXA-24/40, OXA-48, and OXA-51. The catalytic serine and carboxylated lysine residues encompassing the STFK motif are highlighted by the yellow box. The S-A/V-V/I motif is represented by the green box and the K-T/S-G motif is highlighted in blue.
