# Supplementary material for: Structural Analysis of The OXA-48 Carbapenemase Bound to A “Poor” Carbapenem Substrate, Doripenem
Source: Antibiotics (Basel). 2019 Sep 11;8(3):145. doi: 10.3390/antibiotics8030145 (PMC6783824; doi:10.3390/antibiotics8030145)
Supplement: Supplementary file 1 [file antibiotics-08-00145-s001.pdf]

Supplemental information

Structural analysis of the OXA-48 carbapenemase bound to a "poor" carbapenem substrate, doripenem

Krisztina M. Papp-Wallace[1-3*,#], Vijay Kumar[1,#], Elise T. Zeiser[2], Scott A. Becka[2], and Focco van den Akker[1*]

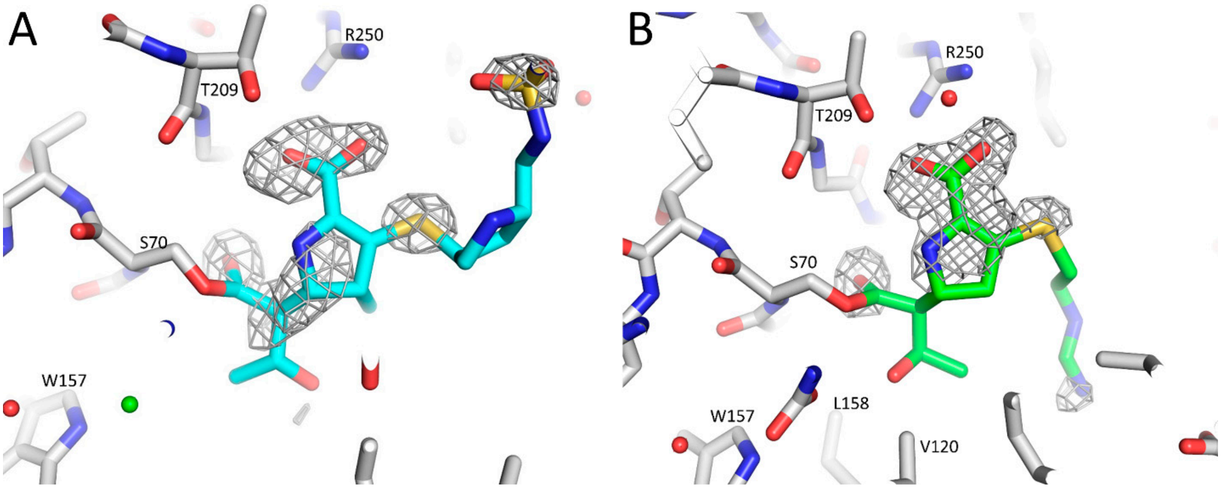

Figure S1. Electron density maps of the active sites of wild-type OXA-48 with doripenem and imipenem bound. To remove ligand bias prior to the map calculation, a 10-cycle refmac crystallographic refinement was carried out with the ligands removed from the coordinates. (A) Unbiased |Fo|-|Fc| Polder omit map of the active site of wild-type OXA-48 showing a poorly ordered doripenem molecule (PDBid = 6P9C molecule A; density contoured at 3 σ). The doripenem ligand was added back to the coordinates prior to the map calculation to allow Polder to adjust the solvent mask. A regular unbiased omit map showed similar results. (B) Unbiased |Fo|-|Fc| omit map of the active site of wild-type OXA-48 showing a poorly ordered imipenem molecule (PDBid = 5QB4 molecule A; density contoured at 3 σ). Electron density in the other molecules in the asymmetric units was equal or worse than the depicted doripenem and imipenem density.

```
CLUSTAL O(1.2.4) multiple sequence alignment


OXA-1           MKNTIHINFAIFLIIANIIYSSASASTDI--------------STVASPLFE--GTEGCF     44
OXA-48          MRV---------LALS-AVFLVASI------IGMPAVAKEWQENKSWNAHFTEHKSQGVV     44
OXA-23          MNKY--F---TCYVVA-SLFLSGCTVQHNLINETP-SQIVQGHNQVIHQYFDEKNTSGVL     53
OXA-24/40       MKKFILP---IFSISI-LVSLSACSSIKTKSEDNF-HISSQQHEKAIKSYFDEAQTQGVI     55
OXA-51          --MNIKT---LLLITS-AIFISACSPYIVTANPNHSASKSDEKAEKIKNLFNEVHTTGVL     54
                                  :   ..                          *    : * .

OXA-1           LLYDASTNAEIAQFNKAKCATQMAPDSTFKIALSLMAFDAEI-IDQKTIFKWDKTPKGME     103
OXA-48          VLWNEN-KQQGFTNNLKRANQAFLPASTFKIPNSLIALDLGVVKDEHQVFKWDGQTRDIA     103
OXA-23          VIQTDK-KINLYGNALSRANTEYVPASTFKMLNALIGLENQK-TDINEIFKWKGEKRSFT     111
OXA-24/40       IIKEGK-NLSTYGNALARANKEYVPASTFKMLNALIGLENHK-ATTNEIFKWDGKKRTYP     113
OXA-51          VIQQGQ-TQQSYGNDLARASTEYVPASTFKMLNALIGLEHHK-ATTTEVFKWDGQKRLFP     112
                ::   . . .       :.     * ****:  :*:.::         :***.   :

OXA-1           IWNSNHTPKTWMQFSVVWVSQEITQKIGLNKIKNYLKDFDYGNQDFSGDKERNNGLTEAW     163
OXA-48          TWNRDHNLITAMKYSVVPVYQEFARQIGEARMSKMLHAFDYGNEDISGN------VDSFW     157
OXA-23          AWEKDMTLGEAMKLSAVPVYQELARRIGLDLMQKEVKRIGFGNAEIGQQ------VDNFW     165
OXA-24/40       MWEKDMTLGEAMALSAVPVYQELARRTGLELMQKEVKRVNFGNTNIGTQ------VDNFW     167
OXA-51          EWEKDMTLGDAMKASAIPVYQDLARRIGLELMSKEVKRVGYGNADIGTQ------VDNFW     166
                 *: : .    *  *.: * *:::::  *   :.: ::  ..:** ::. :      : . *

OXA-1           LESSLKISPEEQIQFLRKIINHNLPVKNSAIENTIENMYLQDLDNSTKLYGKTGAGFTAN     223
OXA-48          LDGGIRISATEQISFLRKLYHNKLHVSERSQ-RIVKQAMLTEANGDYIIRAKTGYSTR--     214
OXA-23          LVGPLKVTPIQEVEFVSQLAHTQLPFSEKVQ-ANVKNMLLLEESNGYKIFGKTGWAMD--     222
OXA-24/40       LVGPLKITPVQEVNFADDLAHNRLPFKLETQ-EEVKKMLLIKEVNGSKIYAKSGWGMG--     224
OXA-51          LVGPLKITPQQEAQFAYKLANKTLPFSPKVQ-DEVQSMLFIEEKNGNKIYAKSGWGWD--     223
                * . ::::  :: .*  .: :  * ..       ::.  : .  ..  : .*:* .

OXA-1           RTLQNGWFEGFIISKSGHKYVFVSALTGNLGSNLTSSIKAKKNAITILNTLNL--   276
OXA-48          IEPKIGWWVGWVELDD-NVWFFAMNMDMPTSD--GLGLRQAI-TKEVLKQEKIIP   265
OXA-23          IKPQVGWLTGWVEQPDGKIVAFALNMEMRSEM--PASIRNEL-LMKSLKQLNII-   273
OXA-24/40       VTPQVGWLTGWVEQANGKKIPFSLNLEMKEGM--SGSIRNEI-TYKSLENLGII-   275
OXA-51          VDPQVGWLTGWVVQPQGNIVAFSLNLEMKKGI--PSSVRKEI-TYKSLEQLGIL-   274
                   : **  *::   . :   *   :          .::        *:   :
```

Figure S2. Multiple sequence alignment using Clustal Ω using wild-type enzymes of OXA-1, OXA-23, OXA-24/40, OXA-48, and OXA-51. The catalytic serine and carboxylated lysine residues encompassing the STFK motif are highlighted by the yellow box. The S-A/V-V/I motif is represented by the green box and the K-T/S-G motif is highlighted in blue.
